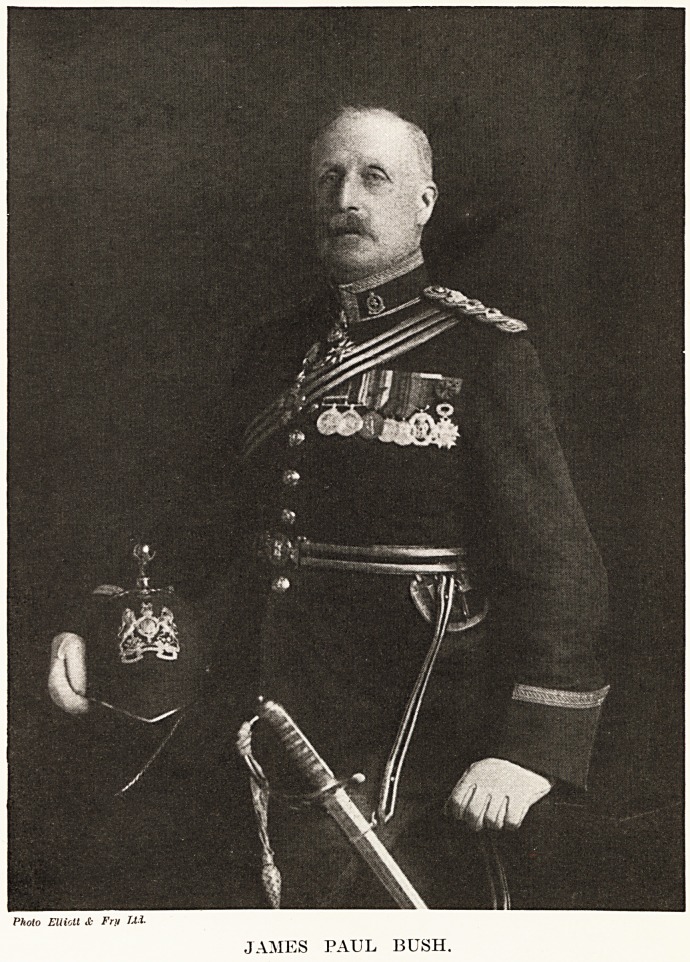# James Paul Bush

**Published:** 1930

**Authors:** 


### Obituary.

### JAMES PAUL BUSH, C.M.G., C.B.E., Ch.M.,

*Consulting Surgeon to the Bristol Royal Infirmary.*

THE death of Colonel Paul Bush, which occurred on the 7th October, at the age of 73, deprived Bristol of a public-spirited citizen, and the medical profession of a popular and distinguished member.

Throughout his life he devoted a great deal of time and energy to public services and to the promotion of various organizations apart from his hospital or private practice, and it was characteristic of him that he retained his interest in many of these projects up to the last, in spite of the prolonged and painful illness which he had borne with such remarkable fortitude.

A member of a well-known Bristol family, Paul Bush was born at Brislington in 1857. After receiving his school education at Clifton College he studied medicine at the Bristol Medical School and Royal Infirmary, and for a short time at University College Hospital, London, before taking the M.R.C.S. in 1881 and the L.S.A. in 1882.

Photo Elliott & Fry Ltd.

JAMES PAUL BUSH.

As a student he soon came into prominence, being good at athletics as well as at his clinical work. For some seasons he captained the medical Rugby and the cricket teams during one of their most successful phases, and at the Infirmary he was awarded both the medical and surgical Suple gold medals. Immediately upon qualifying, Bush began a series of residential posts at the Infirmary, where he was S.R.M.O. when, in 1885, he was appointed Assistant Surgeon. In 1889 he became full Surgeon, and in 1913 Hon. Consulting Surgeon. His active career began just as modern surgery was in its infancy—it being in 1880 that Lister came to the Infirmary and demonstrated his antiseptic technique—and Bush, with his alertness and appreciation of detail, was quick to make himself master of the new method, and from the first he proved himself to be well equipped with the qualities essential to success in the craft, possessing a faculty for combining speed with gentleness and neatness in operating, and being endowed with exceptionally sound judgment and clinical acumen. He was, in fact, a born surgeon, and he was careful to keep pace with any new developments which might appeal to him as progressive. His skill as a surgeon and his consideration for his patients combined to make him extremely popular, and soon he found his time fully occupied, especially as he had many ties in addition to his clinical practice. Being keenly interested in all local medical matters, he served willingly upon a variety of organizing bodies, whether concerned with hospital administration or medical charity, and on several occasions he acted as treasurer, in which capacity he showed marked ability, as in the case of the successful Zoo carnival in 1899, when a sum of £1,480 was handed over to the Infirmary. In connection with these committees it was inevitable that a man of his tenacity of purpose should not infrequently find himself at variance with his colleagues, but it was characteristic of him that he would never allow such differences of opinion to mar friendly relations.

Colonel Bush had always been attracted by military routine and discipline; he served for some years as surgeon to the 1st Volunteer Battalion, Bristol Rifles, at one time commanded by his father. It was no great surprise, therefore, to his friends to learn that he had decided to serve with the troops in South Africa. He went out in February, 1900, as Senior Surgeon to the Princess Christian Hospital, attached to the Natal Field Force, under the command of Major Mathias, R.A.M.C. On arrival at Durban, Bush, with his usual thoroughness, went to a great deal of trouble in selecting a suitable site for the hospital, and was fortunate in obtaining at Pinetown Bridge a position near the Natal railway with its own small, but constant water supply. When the hospital was opened it was quickly filled with officers and men suffering chiefly from typhoid and dysentery. Bush was greatly impressed by the severe degree of illness shown by these patients and took endless pains to do all that was possible, from the clinical or administrative point of view, to lighten their burden. The hospital was referred to in laudatory terms by Lord Roberts in one of his despatches. Colonel Bush left South Africa in the autumn of 1900, acting as P.M.O. in the hospital ship *Lismore Castle* on the homeward journey. He was subsequently awarded the South African Medal with clasps and invested with the order of St. Michael and St. George.

Eight years later, in 1908, Bush undertook the more onerous task of forming, under the new Territorial scheme, the 2nd Southern General Hospital with *à la suite* staff. The first two years of this undertaking involved an enormous amount of work in organizing every detail of the unit in order that it might be ready at any time for immediate mobilization, but Bush was indefatigable, working often into the early hours of morning; few can know the trouble he took to make his unit efficient. He was deservedly proud of the result, for when war broke out in 1914 he was able immediately to mobilize a thoroughly well-equipped and fully-staffed hospital of 520 beds, accommodated partly in the new block of the Infirmary and partly in the newly-built Poor Law Institution at Southmead. As time went on and a large number of subsidiary hospitals were opened within his area his responsibilities grew considerably, but by dint of his methodical organization he was able to keep all departments working smoothly, and to run his whole unit with such success as to elicit a warm expression of appreciation from the Director-General, Sir Alfred Keogh. There was, among his many innovations, one which proved of exceptional value. It consisted of an information bureau, and it soon became a most efficient channel through which to trace the whereabouts of patients in England. In 1917 he went to France in command of the 56th General Hospital, with a staff drawn from the 1st and 2nd Southern General Hospitals in Birmingham and Bristol. The camp at Etaples appealed to Colonel Bush as convenient from an administrative point of view, and he proceeded to erect his hospital in spite of almost daily orders to desist, but marquee after marquee mysteriously went up until eventually the authoities left him alone. He soon had his beds ready and occupied ; his administration was skilful and humane, being aimed always at executive efficiency and patients' comfort. It is not surprising that the arrangements he devised for providing almost unlimited hot baths, even when under canvas, were especially commended. Unfortunately this camp, situate as it was, in the midst of training camps and close to a strategically important railway, came in for very heavy bombing in 1918, and the shock consequent on a succession of these terrifying night ordeals produced an effect upon Colonel Bush from which he never completely recovered.

While in France in 1918 he had the honour of being appointed Deputy-Lieutenant for the County of Gloucester, and in the autumn of the same year, after returning to England, he became A.D.M.S. of No. 2 Area, Southern Command.

After the war he was retired with the rank of Colonel. In recognition of his services he was invested with the Order C.B.E., and by the King of the Belgians he was appointed an Officer of the Crown of Belgium.

The busy nature of Bush's life becomes obvious when we reflect that, in addition to the foregoing, he had many other activities. He was Chief Surgeon to the Bristol Police, Surgeon to the Bristol Post Office, and Consulting Surgeon to the Almondsbury Memorial and the Pontypool Hospitals. In Bristol Medical School and University College he was respectively Demonstrator in Anatomy and Lecturer in Operative Surgery, and for many years a member of the College Council, and soon after the University was founded he received the honorary degree of Ch.M. He was a member of Council of the International Surgical Society, he had held the post of President of the Bath and Bristol Branch of the B.M.A., and had been successively Secretary and President of the Bristol Medico-Chirurgical Society, of which he was made an honorary member in 1928. He was a Vice-President and member of Council of Epsom College, to which he had for many years been a most diligent and successful Local Secretary. In 1887 Bush was Worshipful Master of the St. Vincent Lodge of Freemasons.

Although Paul Bush was a man of such widespread activities and responsibilities, he retained to the last a keen interest in and devotion for his old medical school and hospital, upon whose traditions his name has become so indelibly impressed. He was warm-hearted by nature and very staunch in friendship. He leaves a widow, a son, and a daughter, with whom we deeply sympathize.

## Figures and Tables

**Figure f1:**